# *Ibα-XYL1* Interfered Expression Decreases Starch Granule Size and Increases Soluble Sugar Content to Improve Steamed Sweetpotato Storage Root Taste

**DOI:** 10.3390/ijms26115015

**Published:** 2025-05-23

**Authors:** Chen Li, Meng Kou, Chang You, Meng Ma, Weihan Song, Wei Tang, Hui Yan, Runfei Gao, Xin Wang, Yungang Zhang, Qiang Li

**Affiliations:** 1 Xuzhou Institute of Agricultural Sciences in Jiangsu Xuhuai District, Key Laboratory of Biology and Genetic Breeding of Sweetpotato, Ministry of Agriculture and Rural Affairs, Xuzhou 221131, China; lichen@jaas.ac.cn (C.L.); 20081003@jaas.ac.cn (M.K.); yclucky2022@126.com (C.Y.); xzsongweihan@163.com (W.S.); 20091002@jaas.ac.cn (W.T.); yanhuijsxz@jaas.ac.cn (H.Y.); grf15852141027@gmail.com (R.G.); xznkywx@163.com (X.W.); zhangyungang@jaas.ac.cn (Y.Z.); 2 School of Life Science, Jiangsu Normal University, Xuzhou 221116, China; brave_ma_2022@163.com

**Keywords:** sweetpotato, xylosidase, starch, soluble sugar, taste

## Abstract

As an important characteristic and horticultural crop in China, sweetpotato can be used as food, industrial raw material, vegetable, and ornamental material. Purple sweetpotato for table use is rich in anthocyanin, which leads to some bitter taste, so it needs further quality improvement. Genetic engineering technology is an effective method to improve crop traits, but there are few reports on genes that can improve sweetpotato sweetness and taste. A xylosidase gene (*Ibα-XYL1*) was cloned from sweetpotato variety ‘Yanshu 25’ with a fragment size of 2796 bp and encoding 932 amino acid sequences. It has a typical transmembrane domain and three functional domains, which are localized at cell membrane. Reduction in *Ibα-XYL1* gene expression had no significant effect on the expansion characteristics and anthocyanin content of sweetpotato storage root (SPSR), but it could up-regulate the expression of sucrose synthesis related genes (*SuS*, *SuPS*) and promote the accumulation of soluble sugar in fresh transgenic SPSR. At the same time, it could up-regulate the expression of genes related to starch synthesis modifications (*GASS*, *SBE*) and starch decomposition (*AMY* and *BAM*), reduce the starch granule size and the starch pasting temperature, promote the conversion of starch to maltose, increase the soluble sugar content, and improve the sweetness and taste of steamed transgenic SPSR. The results are of great significance for quality improvement of sweetpotato.

## 1. Introduction

Sweetpotato (*Ipomoea batatas* (L.) Lam.) is an important characteristic crop and horticultural crop in China. Its stem and leaf can be eaten as vegetable, and its storage root can be used as food and processing raw material. At the same time, some special sweetpotato varieties can be used as landscaping material. Due to its plentiful uses, it is widely planted in various regions of China. PFSP is one type of sweetpotato. It is rich in anthocyanin and has antioxidant activity and is highly popular with people [1]. However, due to the influence of anthocyanin, PFSP has a bitter taste, which makes it unpleasant to people. Therefore, improving the taste of PFSP has become an important research direction.

Soluble sugar content is an important index to evaluate SPSR taste. SPSR mainly contains four soluble sugars: glucose, fructose, sucrose, and maltose [2]. The soluble sugar in fresh SPSR has a certain effect on the final taste, but the main factor is maltose produced by starch gelatinization in the ripening process [3]. The starch gelatinization characteristics are mainly affected by starch structure, and small starch granules are easier to gelatinize and decompose, so they have a lower gelatinization temperature [4]. Related studies also show that SPSR gelatinization temperature is inversely proportional to the maltose content, and the lower the gelatinization temperature, the more maltose will be produced [5]. Overexpression of sucrose non-fermentation-associated protein kinase-1 (*SnRK1*) can increase starch content, decrease amylose ratio, increase granule size, and increase crystallinity and gelatinization of transgenic sweetpotato [6], but there are no results about whether it had any effect on soluble sugar content.

In addition, maltose content is also affected by amylase, especially β-amylase [7,8]. Under the same conditions, higher amylase activity and thermal stability will produce more maltose [5]. However, when maltose content increases to a certain amount, it will no longer increase, indicating that there are other limiting factors affecting maltose synthesis, such as substrate restriction and feedback of high concentration products [9]. Since β-amylase cannot break down ungelatinized starch grains, starch gelatinization becomes a necessary and limiting condition for amylase to decompose starch into maltose. In addition, some reports found that vacuolar invertases (*IbINV*) [10] and fructose-1,6-bisphosphate aldolase (*IbFBA5*) [11] can increase SPSR soluble sugar content, but whether it affects SPSR taste has not been reported.

Alpha-xylosidase (α-XYL) is a member of the glycoside hydrolase 31 family. Like the other members, α-xylosidase can cut α-xylose residues from the non-reducing ends of the glycoside chain. α-xylosidase is an important enzyme in oligosaccharide degradation and was discovered in some bacteria, such as *Sulfolobus solfataricus* [12], *Cellvibrio japonicus* [13], *Aspergillus niger* [14], etc. It is the first report that α-xylosidase-coding proteins have high activity against α-xylosides, especially against xylo-oligosaccharides, but hardly hydrolyzes the α-glucoside bond in rice [15]. However, in *Arabidopsis thaliana*, α-xylosidase plays an important role in xylan remodeling, maintaining cell wall integrity, and seed germination [16], and is also involved in polysaccharides decomposition, especially xylo-oligosaccharides [17]. When *AtXYL1* was introduced in a suitable expression vector and used to transform *Saccharomyces cerevisiae*, significantly higher α-xylosidase activity was detected in the yeast cells. α-glucosidase activity was also increased in the transformed cells, although to a lesser extent [18]. *Arabidopsis* mutant seeds lacking *TRG1/XYL1* showed insensitivity to adverse dormancy environment, decreased elasticity of elongated stems, and decreased free xylooligosaccharides content [16]. However, the function of xylosidase in SPSR is still unknown, and whether it affects soluble sugar accumulation has not been reported. Therefore, we cloned an *Iba-XYL1* and verified its function in SPSR using genetic transformation. The research results will provide new ways for quality improvement of SPSR.

## 2. Results

### 2.1. Cloning, Sequence and Expression Pattern Analysis of Ibα-XYL1

**Figure 1:**
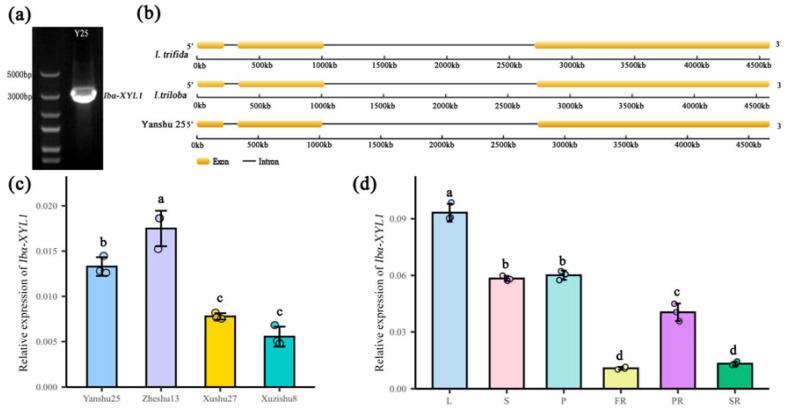
Cloning, sequence and expression pattern analysis of *Ibα-XYL1*. (**a**) The gel map of *Ibα-XYL1* amplified using sweetpotato variety ‘Yanshu 25’ as template. (**b**) Gene structure analysis (**c**) Relative expression levels of *Ibα-XYL1* in four different saccharification varieties. (**d**) Relative expression levels of *Ibα-XYL1* in different tissues. L, S, P, FR, PR, and SR represent leaf, stem, petiole, fibrous root, pencil root, and storage root, respectively. The three dots on each column in (**c**,**d**) represent the results of three independent biological experiments. Each bar represents the mean ± SD. Lowercase letters on the bars indicate analysis of variance (*p* < 0.01).

The unigene sequence obtained from the saccharification-related transcriptome data [19] was compared with sweetpotato GARDEN (http://sweetpotato-garden.kazusa.or.jp/index.html). The SPSR cDNA of variety ‘Yanshu 25’ was used as a template to amplify the target gene fragment (Figure 1a). After sequencing and alignment, the unigene was annotated as a xylosidase encoding gene, which was homologous to *Itfα-XYL1*, and was therefore named *Ibα-XYL1*. The *Itfα-XYL1* sequence comes from a public database (https://sweetpotato.uga.edu/, accessed on 20 October 2019). *Ibα-XYL1* fragment is 2796 bp in size and encodes 932 amino acids. At the same time, DNA sequence of *Ibα-XYL1* was cloned and gene structure was analyzed. It was found that its gene structure was consistent with that of two wild sweetpotato species, *Ipomoea trifida* and *Ipomoea triloba*, and contained two introns and three exons (Figure 1b).

In addition, RT-qPCR was used to detect the expression of *Ibα-XYL1* in four different saccharification characteristics of sweetpotato varieties and the expression in different tissues of ‘Yanshu 25’. The results showed that the expression levels in storage roots of high-saccharification varieties ‘Yanshu 25’ and ‘Zheshu 13’ were significantly higher than those of low-saccharification variety ‘Xushu 27’ and ‘Xuzishu 8’ (Figure 1c), indicating that it may be related to sweetpotato saccharification. In addition, the expression level of *Ibα-XYL1* in aboveground tissues, especially in leaves, is higher than that in underground tissues (Figure 1d).

### 2.2. Ibα-XYL1 Protein Structure, Properties Analysis, and Subcellular Localization

**Figure 2:**
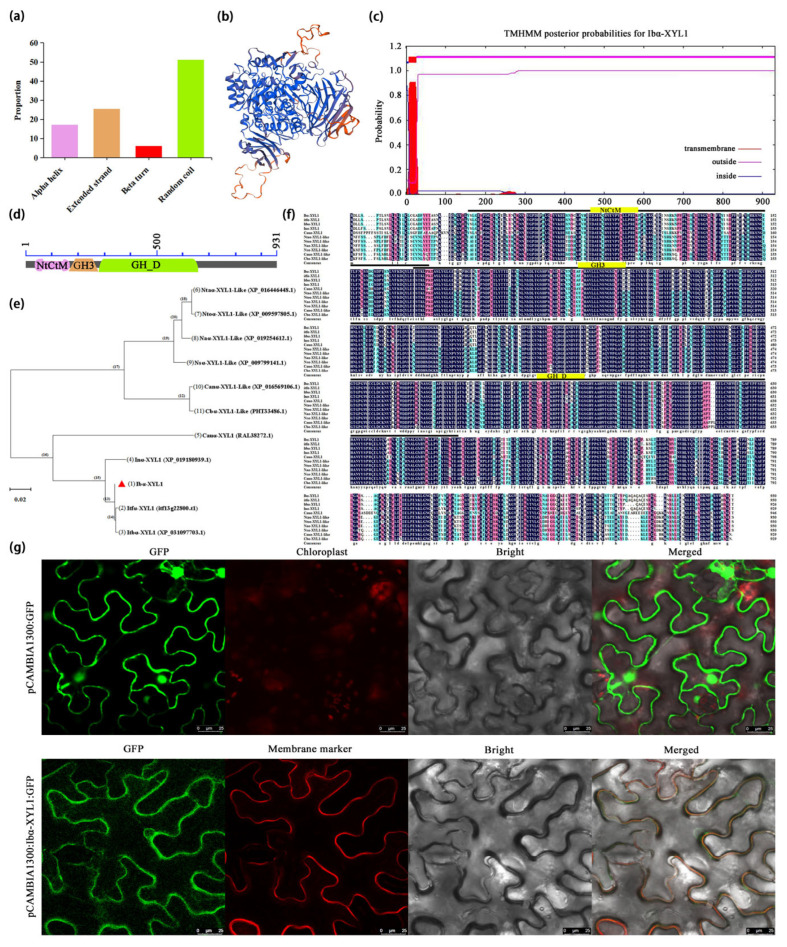
Ibα-XYL1 protein structure, properties and subcellular localization. (**a**) Secondary structure prediction. (**b**) Three-dimensional structure prediction. In the figure, blue represents hydrophilic amino acids and red represents hydrophobic amino acids. (**c**) Transmembrane domain prediction. (**d**) Conserved domains prediction. (**e**) Phylogenetic analysis of α-XYL1. Phylogenetic tree analysis was performed using the minimum evolutionary distance method. The 10 species with high homology in the (**e**) are *Ipomoea trifida* (itf13g22800.t1), *Ipomoea triloba* (XP_031097703.1), *Ipomoea nil* (XP_019180939.1), *Cuscuta australis* (RAL38272.1), *Nicotiana tabacum* (XP_016446448.1), *Nicotiana tomentosiformis* (XP_009597805.1), *Nicotiana attenuata* (XP_019254612.1), *Nicotiana sylvestris* (XP_009799141.1), *Capsicum annuum* (XP_016569106.1), and *Capsicum baccatum* (PHT33486.1), respectively. The red triangles in the figure indicate the genes in this study. (**f**) Multiple alignment analysis of α-XYL1 amino acid sequences. The 10 species in the (**f**) are the same as (**e**). (**g**) Subcellular localization. The image scale is 25 μm, the excitation wavelength of EGFP is 488 nm, the emission wavelength is 510 nm, the excitation wavelength of cell membrane maker is 587 nm, and the emission wavelength is 610 nm.

The *Ibα-XYL1* sequence was input into NCBI to obtain the full-length ORF sequence and its corresponding amino acid sequence. The secondary structure of Ibα-XYL1 was predicted using the online tool SOPMA. The results showed that the protein mainly has four secondary structures: α-helix, β-turn, extended chain, and random coil (Figure 2a). Three-dimensional model prediction analysis of Ibα-XYL1 found that it had the highest similarity with α-glucosidase in the same family (Figure 2b). The transmembrane domain prediction analysis showed that the Ibα-XYL1 had a typical transmembrane structure at amino acids 7–26 (Figure 2c). Ibα-XYL1 mainly contains three functional domains: NtCtM (47–182 aa), GH3 (175–293 aa), and GH_D (293–673 aa) (Figure 2d). Comparing the 10 most similar species sequences from the NCBI, and performing phylogenetic tree analysis, it was found that Ibα-XYL was most closely related to two wild species of sweetpotato (*Ipomoea trifida* and *Ipomoea triloba*), followed by *Ipomoea nill* of the same family (Figure 2e). Multiple sequence alignment found that the sequences of 10 species were very conservative in three functional domains (Figure 2f).

The recombinant Ibα-XYL1::EGFP plasmid and cell membrane maker were transiently transformed into tobacco leaves by injecting into the lower epidermis. The fluorescence was observed by laser confocal microscopy at 488 nm, with pCAMBIA1300::GFP as a control. Under different excitation light, the Ibα-XYL1::EGFP fusion protein emits green fluorescence, while the cell membrane localization marker emits red fluorescence. When two images are merged, it is found that red fluorescence and green fluorescence are fused together to form a yellow signal, indicating that Ibα-XYL1::EGFP fusion protein is localized on the cell membrane (Figure 2g). The previous transmembrane prediction also found that Ibα-XYL1 has a transmembrane structure and the subcellular localization results both indicate that Ibα-XYL1 is a membrane protein.

### 2.3. Acquisition, Phenotypes, Yield Traits, and Anthocyanin Content Analysis of Transgenic SPSR

**Figure 3:**
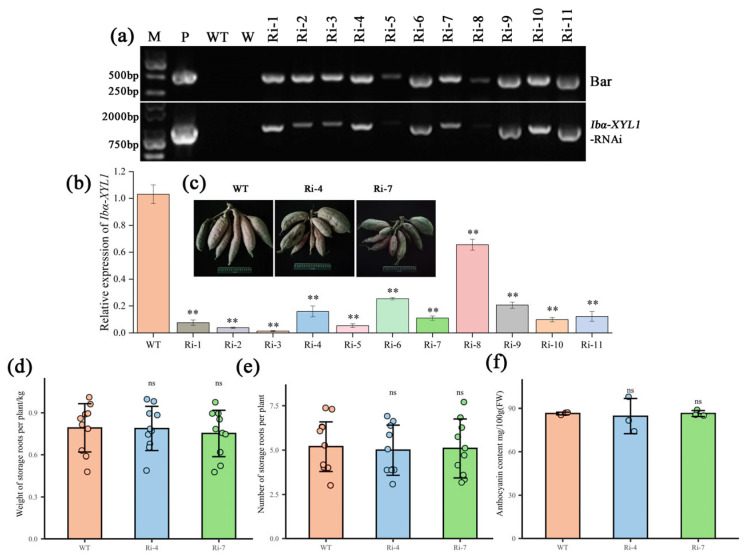
Acquisition, phenotypes, yield traits, and anthocyanin content analysis of transgenic SPSR. (**a**) PCR detection of transgenic lines. In the gel images, P, WT, and W represent positive control, wild-type control, and water, respectively. Each transgenic line was double detected using primers for the selection marker *bar* gene and primers for the vector. (**b**) Relative expression analysis of *Ibα-XYL1* gene in transgenic lines. Each bar represents the mean ± SD and “**” represents significant difference compared with WT (*p* < 0.01). (**c**) Storage roots formation phenotypes. The WT used in this experiment were all plants that were redifferentiated into seedlings using callus tissue of ‘Xuzishu 8’ which was carried out simultaneously with the transgenic process. (**d**) Weight of storage roots per plan. (**e**) Number of storage roots per plan. (**f**) Anthocyanin content in WT and transgenic lines. In the figure, "ns" means no significant difference.

The *Ibα-XYL1*-RNAi vector was stably transformed into ‘Xuzishu 8’ callus using *Agrobacterium tumefaciens*-mediated method. After hygromycin resistance screening and PCR detection, 11 transgenic lines were obtained (Figure 3a). RNA was extracted from transgenic SPSR and the expression of *Ibα-XYL1* gene was detected. It was found that compared with WT, the expression of *Ibα-XYL1* in transgenic lines was significantly down-regulated (Figure 3b). Based on the phenotype and gene expression level of the transgenic lines, two transgenic lines (Ri4 and Ri7) were selected. After a growth cycle of planting, it was found that the storage roots of transgenic lines and WT could expand normally (Figure 3c), and no significant difference was found in storage roots-forming habits, especially the number and weight of storage roots per plant (Figure 3d,e). At the same time, there was no significant difference in anthocyanin content in storage roots (Figure 3f). These results indicate that interference with the expression of *Ibα-XYL1* has no obvious effect on SPSR yield and anthocyanin content.

### 2.4. Interference with the Expression of Ibα-XYL1 Decreases Starch Granule Size and Increases Soluble Sugar Content of Fresh Transgenic SPSR

**Figure 4:**
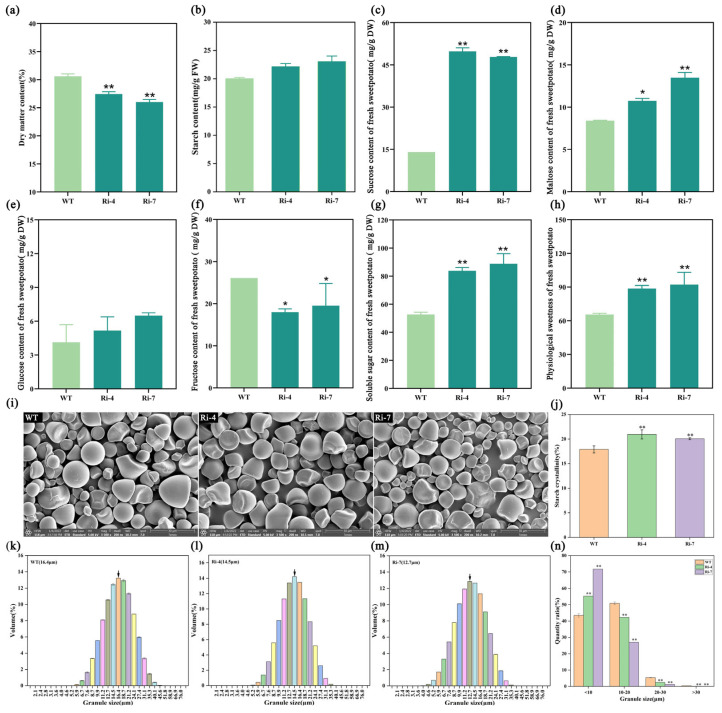
Quality traits analysis of fresh transgenic SPSR. (**a**) Dry matter content. (**b**) Starch content. (**c**–**f**) Sucrose, maltose, glucose, and fructose content. (**g**) Soluble sugar content. (**h**) Physiological sweetness. (**i**) Scanning electron microscopy of starch granules. The picture scale is 30 μm. (**j**) Starch crystallinity. (**k**–**m**) The proportion of different sizes starch granules. The arrow indicates the highest peak. (**n**) The number of different sizes starch granules. Each bar represents the mean ± SD, “*” represents significant difference compared with WT (*p* < 0.05) and “**” represents significant difference compared with WT (*p* < 0.01).

The quality traits of transgenic lines were further determined. The results showed that dry matter content of transgenic lines was significantly lower than that of WT (Figure 4a), and starch content increased slightly (Figure 4b). Further analysis of soluble sugar components showed that sucrose (Figure 4c) and maltose (Figure 4d) content increased significantly, glucose (Figure 4e) content increased slightly, while fructose (Figure 4f) content decreased significantly. At the same time, soluble sugar content in fresh SPSR increased significantly (Figure 4g), resulting in an increase in physiological sweetness (Figure 4h). Since soluble sugar and starch belong to the same metabolic pathway, we analyzed the starch structure in fresh transgenic SPSR. The results showed that starch granules of transgenic lines were significantly smaller than those of WT (Figure 4i), and starch crystallinity was increased significantly (Figure 4j). Further starch granules volume distribution analysis showed that 16.4 μm starch granules accounted for the highest proportion in WT (Figure 4k), while 14.5 μm and 12.7 μm starch granules accounted for the highest proportion in the two transgenic lines (Figure 4l,m), indicating that starch granule size of the transgenic line was significantly reduced. Similarly, by counting different sizes of starch granules number, it was further confirmed that starch granules smaller than 10 μm in transgenic lines were significantly higher than that in WT (Figure 4n). The above results showed that interference with the expression of *Ibα-XYL1* promoted soluble sugar content, especially maltose and sucrose, improved the physiological sweetness of fresh SPSR, and changed starch granules size and crystallinity.

### 2.5. Interference with the Expression of Ibα-XYL1 Improved Soluble Sugar Content, Sweetness, and Taste of Steamed Transgenic SPSR

**Figure 5:**
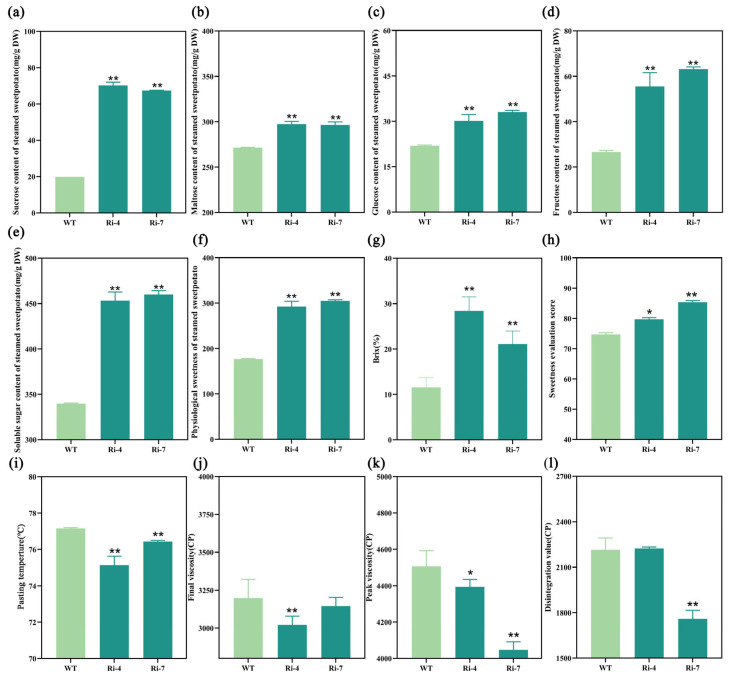
Quality traits and starch gelatinization characteristics of steamed transgenic SPSR. (**a**–**d**) Sucrose, maltose, glucose, fructose content. (**e**) Soluble sugar content. (**f**) Physiological sweetness. (**g**) Sweetness measured by brix meter. (**h**) Sweetness evaluation scores. (**i**–**l**) Starch pasting temperature, final viscosity, peak viscosity and disintegration value analysis. Each bar represents the mean ± SD, “*” represents significant difference compared with WT (*p* < 0.05) and “**” represents significant difference compared with WT (*p* < 0.01).

We all know that changes in starch structure will inevitably lead to changes in starch gelatinization characteristics, affect starch decomposition and the production of soluble sugar, and lead to differences in the taste of steamed SPSR. Therefore, we further determined the soluble sugar content after being steamed. The results showed that sucrose(Figure 5a), maltose(Figure 5b), glucose(Figure 5c), and fructose(Figure 5d) contents of steamed transgenic SPSR were significantly higher than those of WT, which led to a sharp increase in soluble sugar content (Figure 5e). However, the content of sucrose, glucose, and fructose after being steamed only slightly increased compared with that in fresh sweetpotatoes, while the content of maltose showed a very significant increase. These results are consistent with relevant literature reports [3], in which the increase in maltose content may be due to the decomposition of starch. At the same time, the physiological sweetness (Figure 5f) and sweetness (Figure 5g) which measured by brix meter steamed of transgenic SPSR were significantly higher than those of WT. Further, the artificial sweetness evaluation score confirmed that the sweetness and taste of the transgenic line were significantly higher than that of WT (Figure 5h). In addition, the pasting temperature (Figure 5i), final viscosity (Figure 5j), peak viscosity (Figure 5k), and disintegration value (Figure 5l) of transgenic lines were significantly lower or low than those of WT. The results showed that changes in starch granules size led to the reduction in gelatinization characteristics, promoted the conversion of starch to soluble sugar, and improved sweetness and taste in steamed transgenic SPSR.

### 2.6. Interference with the Expression of Ibα-XYL1 Affects the Expression of Genes Related to Starch and Sucrose Metabolic Pathways

**Figure 6:**
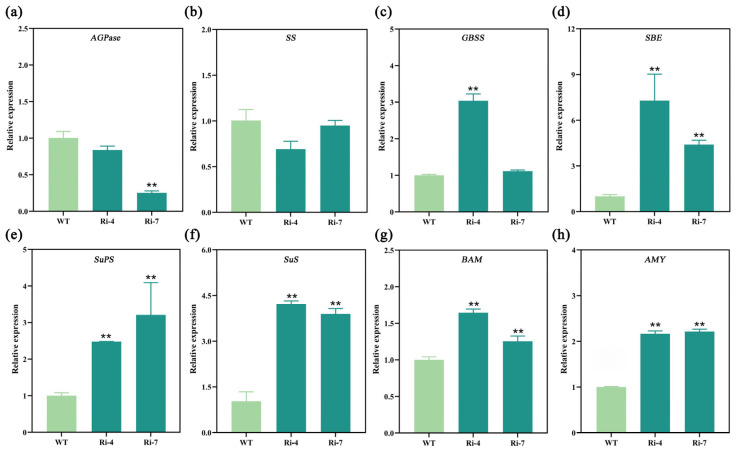
Expression analysis of genes related to starch metabolism. (**a**–**h**) Analysis of the relative expression levels of adenosine diphosphate glucose pyrophosphorylase gene (*AGPase*), starch synthase gene (*SS*), granulated starch synthase gene (*GBSS*), starch branching enzyme gene (*SBE*), sucrose phosphate synthasee gene (*SuPS*), sucrose synthetase gene (*SuS*), β-amylase gene (*BAM*), and α-amylase gene (*AMY*) genes in transgenic lines and WT storage roots. Each bar represents the mean ± SD and “**” represents significant difference compared with WT (*p* < 0.01).

RT-qPCR was used to analyze the expression levels of genes related to starch and sucrose metabolism in transgenic lines and WT storage roots. It was found that the expression levels of adenosine diphosphate glucose pyrophosphorylase gene (*AGPase*) (Figure 6a) and starch synthase gene (*SS*) (Figure 6b) related to starch synthesis were significantly decreased, the expression levels of granulated starch synthase gene (*GBSS*) (Figure 6c) and starch branching enzyme gene (*SBE*) (Figure 6d) related to starch synthesis modification were significantly increased, and the expression levels of sucrose phosphate synthasee gene (*SuPS*) (Figure 6e) and sucrose synthetase gene (*SuS*) (Figure 6f) related to sucrose synthesis were significantly increased. At the same time, β-amylase gene (*BAM*) (Figure 6g) and α-amylase gene (*AMY*) (Figure 6h) related to the breakdown of glucose chain were significantly increased after starch gelatinization. The results showed that *Ibα-XYL1* could affect the expression of genes related to starch and sucrose metabolic pathways to regulate changes in starch structure and sugar content.

## 3. Discussion

As an important characteristic crop, sweetpotato plays an important role in ensuring food safety and enriching food types [20]. With the improvement of people’s living standards, the requirements for food quality, especially taste and nutrition, are getting higher and higher. As an important type of table use sweetpotato, PFSP can meet the nutritional function requirements, but the taste is not good, so it is urgent to improve the taste quality. Therefore, in this paper, we cloned a xylosidase gene from ‘Yanshu 25’, a high-saccharidation sweetpotato variety, and genetically transformed it into a PFSP variety ‘Xuzishu 8’. We found that *Ibα-XYL1* interfered expression can effectively increase soluble sugars content in SPSR and improve PFSP taste.

### 3.1. Interference with the Expression of Ibα-XYL1 Did Not Affect Yield Traits and Anthocyanin Content in SPSR

The storage roots-forming habits directly determine the yield and economic characteristics of sweetpotato. Because *Ibα-XYL1* is a member of the glycoside hydrolase 31 (GH31) family, all GH31 enzymes can cut the carbohydrate portion from the end of the substrate, but the size and type of substrate (e.g., starch, glycoprotein, etc.) varies from enzyme to enzyme [15]. Previous reports have found that *Atα-XYL1* in *Arabidopsis thaliana* has the functions of xylan remodeling, maintaining the integrity of cell wall, and regulating seed germination, etc. [16]. The absence of *α-XYL1* will cause insensitivity to adverse dormancy environment, and the elasticity of elongated stems will decrease, etc. [16], but this phenomenon is not found interfering in transgenic sweetpotato plants, and the storage roots of transgenic lines can germinate normally and be used as breeding materials. There was no significant difference in the number and habit of storage root formation between transgenic lines and WT (Figure 3d,e). It may be that there are some differences between sweetpotato and *Arabidopsis* seed propagation as asexual. Comparing the amino acid sequences of *Arabidopsis* (Locus: AT1G68560) and sweetpotato α-xylosidase, it was also found that the amino acid sequences were only 71.46% similar (Figure S1), indicating that the two protein structures may be quite different. Previous experiments in vitro found that Atα-XYL1 may have some glucosidase activity [18], but in this study it was found that the glucose content in transgenic fresh sweetpotatoes did not change significantly (Figure 4e), indicating that α-xylosidase may not have glucosidase activity in sweetpotato storage roots. At the same time, the substrate specificity and enzyme kinetics of α-xylosidase in sweetpotato storage roots need to be further studied.

Anthocyanins are crucial nutrients in PFSP and play an important role in human health. While improving the taste of PFSPs, we do not want to reduce the anthocyanin content. Interestingly, through cross-section observation (Figure S2) of *Ibα-XYL1* interfered expression transgenic SPSR and determination of anthocyanin content (Figure 3f), it was found that the anthocyanin content did not change significantly, indicating that the gene had no effect on anthocyanin synthesis and accumulation.

### 3.2. Interference with the Expression of Ibα-XYL1 Decreases Starch Granule Size and Change Starch Gelatinization Properties

Starch synthesis is a complex process that includes not only the synthesis of upstream amylopectin and amylose, but also the modification of amylopectin chain length and branches, and the reorganization and crystallization of starch granules. This study found that there was a slight increase in starch content (Figure 4b) of *Ibα-XYL1* interfered expression transgenic SPSR, but it did not reach a significantly level, although expression levels of genes (*AGPase*, *SS*) related to starch synthesis was down-regulated slightly (Figure 6a,b). There is a direct relationship between the expression levels of *AGPase*, *SS* and starch content [21,22,23]. In fact, we can find that the down-regulation level of these two genes is not at a very significant level, which may be the main reason why starch content has not significantly decreased. More interestingly, the expression level of genes related to starch synthesis modification (Figure 6c,d) has been significantly up-regulated. Relevant reports show that *GBSS* and *SBE* are mainly used to modify the chain length of amylopectin and amylose, which have little effect on the content but can change starch structure [24,25]. Therefore, we analyzed the starch granules of *Ibα-XYL1* interfered expression transgenic SPSR and found that the starch granules size decreased and the number in small starch granules increased (Figure 4i–n). With the decrease in starch granule size, gelatinization temperature decreased, and gelatinization temperature was inversely proportional to the maltose content [5]. Our experimental results also confirm this conclusion (Figure 5b,i).

### 3.3. Interference with the Expression of Ibα-XYL1 Increases the Soluble Sugar Content of Fresh and Steamed SPSR, and Improves the Sweetness and Taste of Steamed SPSR

Through the analysis of the soluble sugar content of fresh and steamed SPSR, it was found that the disaccharides in fresh SPSR increased significantly (Figure 4c,d), and all soluble sugars in steamed SPSR increased significantly (Figure 5a–d). The reason for the increase in sugar content fresh SPSRs may be due to the significant up-regulation of the expression level of genes (*SuPS* (Figure 6e) and *SuS* (Figure 6f)) related to sucrose metabolism. The relevant literature has confirmed that up-regulated expression of *SuPS* and *SuS* can significantly increase sucrose content. At the same time, the reduction in starch granules size leads to a decrease in starch gelatinization temperature and promotes the conversion of starch to maltose [5,26]. In addition, the significant increase in amylase gene (*BAM*, *AMY*) expression (Figure 6g,h) promotes the decomposition of starch and the accumulation of soluble sugar in steamed SPSR [8].

Interestingly, we used online tools (https://cn.string-db.org/cgi/input?sessionId=lJLa6T80jCeQ&input_page_show_search=on) to predict the interaction of Ibα-XYL1 proteins and found that α-XYL1 proteins can interact with AMY2 and AMY3 [27,28], as well as β-galactosidase (BGAL10) and fucosidase (FUC95A) (Figure S3). At the same time, we also analyzed the interaction proteins of Ibα-XYL1 by CO-IP-MS. In the results, we screened proteins related to sucrose synthesis and decomposition, such as sucrose synthase (SuS), sucrose invertase (INV), sucrose phosphate synthase (SuPS), etc. At the same time, we also screened SBE, which are related to starch synthesis and modification (Table S1). The above results support the interaction between Ibα-XYL1 and enzymes related to starch metabolism in a certain sense, but more experimental support is needed.

### 3.4. Molecular Model Predictions of Interference with the Expression of Ibα-XYL1 Regulation for Improving SPSR Taste

**Figure 7:**
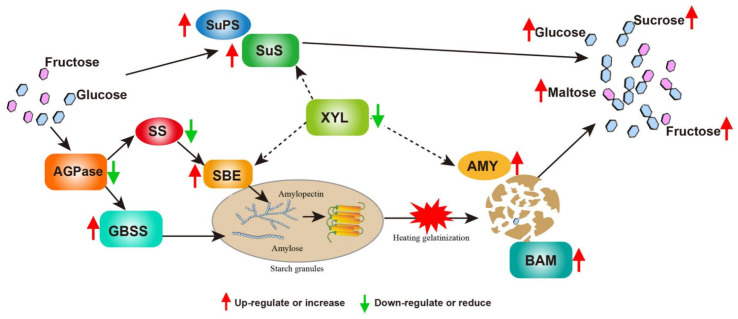
Molecular model predictions of *Ibα-XYL1* interfered expression regulation for improving SPSR taste.

Based on the above experimental results and discussion, we established a molecular model in which *Ibα-XYL1* regulates starch metabolism in SPSR and affects the final taste, as shown in Figure 7. Firstly, *Ibα-XYL1* interfered expression promotes the up-regulated expression of sucrose synthase-related genes and promotes the synthesis and accumulation of soluble sugar content in fresh SPSR. Secondly, the change in monosaccharide content, especially fructose content may affect the expression level of *AGPase* and *SS*. At the same time, the interference of *Ibα-XYL1* expression promoted the up-regulated expression of genes related to starch synthesis modification, such as *GBSS* and *SBE*, and changed the size and crystallinity of sweetpotato starch granules in fresh SPSR. Finally, the decrease in starch granule size leads to the decrease in starch pasting temperature, and the interference of *Ibα-XYL1* expression up-regulated the expression of *AMY* and *BAM*, accelerated starch decomposition, produced more disaccharides (maltose and sucrose), and finally affected the sweetness and taste of steamed SPSR.

## 4. Materials and Methods

### 4.1. Plant Materials

Sweetpotato varieties ‘Xushu 27’ and ‘Xuzishu 8’ (low-saccharification variety), ‘Yanshu 25’ (high-saccharification variety), and ‘Zheshu 13’ (high-saccharification variety) were selected as experimental materials for analyzing the expression of *Ibα-XYL1*, which were planted at the experimental base of Xuzhou Academy of Agricultural Sciences at the same time, and ‘Yanshu 25’ was also utilized to clone the *Ibα-XYL1* in this study. PFSP variety ‘Xuzishu 8’ was used as a receptor material for *Ibα-XYL1* transformation and functional analysis.

Yanshu 25, Zheshu 13, Xushu 27, and Xuzishu 8 were planted in the same community. Each community planted 20 plants and set up 3 replicates. Samples were taken 130 days after planting, and 3 plants were randomly dug out from each community, making a total of 9 plants. The harvested storage roots were washed and dried before using them to extract RNA.

### 4.2. Ibα-XYL1 Cloning and Expression Pattern Analysis

The total RNA was extracted by RNA extraction kit (Quick RNA Isolation Kit, Beijing Huayueyang Biological Technology Co., LTD., Beijing, China, Cat.:0416-50-GK) from ‘Yanshu 25’ SR and the first cDNA was synthesized by reverse transcription kit (ReverTra Ace^®^ qPCR RT Master Mix with gDNA Remover, TOYOBO LIFE SICENCE, Osaka, Japan, Code:FSQ-301) for cloning *Ibα-XYL1*. The cloned fragments were purified and ligated into the pEASY^®^-Blunt Cloning Vector (pEASY^®^-Blunt Cloning Kit, Beijing transgen Biotechnology Co., Ltd., Beijing, China, Cat.:CB101-01) and sequenced to check its correctness. At the same time, the genomic sequence of *Ibα-XYL1* was cloned using the ‘Yanshu 25’ leaves DNA as a template. The genetic structure of *Ibα-XYL1* was analyzed using the online software GSDS2.0 (https://gsds.gao-lab.org/).

RT-qPCR was used to analyze *Ibα-XYL1* expression in different tissues and varieties. Different tissue samples were derived from ‘Yanshu 25’ plants at 130 days after planting (DAP). Different sweetpotato varieties storage roots were derived from plants at 130 DAP. All RT-qPCR analyses were performed using the ABI QuantStudio 6 Flex Q6 system (Thermo Fisher Scientific Inc., Waltham, MA, USA) and 2 × Q3 SYBR qPCR Master Mix (Tolo Biotech Co., Ltd., Shanghai, China) according to the manufacturer’s instructions. Specific primers are shown in Table S1. *IbARF* was used as the internal reference gene [29], and the 2^−ΔΔCT^ method was utilized to analyze the relative expression levels [30].

### 4.3. Ibα-XYL1 Protein Sequence, Structure, Properties Analysis, and Subcellular Localization

The secondary structure was analyzed with the online tool SOPMA (https://npsa-prabi.ibcp.fr/cgi-bin/npsa_automat.pl?page=npsa_sopma.html#opennewwindow), and the three-dimensional model of *Ibα-XYL1* was predicted with the online software SWISS-MODEL (https://swissmodel.expasy.org/; Version 5.2.2.0). The online tool TMHMM (https://services.healthtech.dtu.dk/services/TMHMM-2.0/; Version TMHMM 2.0) and SMART (http://smart.embl-heidelberg.de/) were used to predict the transmembrane domain and conserved domains of Ibα-XYL1. The MEGA7.0 software was used to perform phylogenetic analysis of α-XYL protein sequences from different plants using the neighbor-joining method. DNAMAN (Version 5.2.2.0; LynnonBiosoft, San Ramon, CA, USA) and BLAST (https://blast.ncbi.nlm.nih.gov/Blast.cgi; Version BLAST+ 2.3.0) were used to align α-XYL protein sequences.

The CDS sequence of *Ibα-XYL1* without stop codon was amplified and ligated to pCAMBIA1300 to construct Ibα-XYL1::GFP expression vector, followed by transformation of Agrobacterium strain GV3101, immersion of Nicotiana benthamiana, and observation of GFP fluorescence under a confocal microscope (TCSSP8; Leica, Wetzlar, Germany). The specific operation steps refer to [31], with pCAMBIA1300::GFP as control.

### 4.4. Transgenic Sweetpotato Plants Production and Phenotypic Analysis

Using pCAMBIA9541 as vector backbone, The Ibα-XYL1-RNAi vector was constructed (Figure S4), and the construction method was referred to Kou [32]. The pFGC5941 vector was digested by double *NcoI* and *SwaI* digestion. The FS-Ibα-XYL1, RS-Ibα-XYL1, and Intron fragments were amplified by PCR and recovered. FS-Ibα-XYL1 and RS-Ibα-XYL1 are complementary sequences, which, together with Intron, form a neck-loop structure. The pFGC5941 digestion product, FS-Ibα-XYL1, RS-Ibα-XYL1, and intron fragments were connected through homologous recombinase to construct an RNAi-Ibα-XYL1 vector. Using ‘Xuzishu 8’ callus as the transformation material, genetic transformation was carried out, and the specific transformation steps were referred to Liu [33].

The transgenic lines and WT were planted in the same community, with 20 plants per variety in each community, and 3 replicates were set up. Sampling began 130 days after planting, and 3 plants were dug out from each community, making a total of 9 plants. The storage roots obtained were used for quality analysis. The number and weight of storage roots per plant of 10 plants were randomly determined for each line, and the storage roots-forming habits of each line were photographed.

### 4.5. Transgenic Sweetpotato Physicochemical Properties Analysis

The obtained sweetpotato storage roots were randomly divided into two groups. One set is used to measure the quality indicators of fresh sweetpotato, and the other set is used to measure the quality indicators of steamed sweetpotato. Fresh samples were sampled, chopped, quickly placed in liquid nitrogen for 30 min, and then placed in a vacuum freeze dryer for freeze-drying. The other group of samples was placed in a steaming box, steamed at 100 °C for 60 min, and then placed in a vacuum freeze dryer for freeze-drying.

The dry matter content was determined by freeze-drying method [34]. The total anthocyanin content of sweet potato tissues was measured in accordance the method proposed by Kou [32] and calculated using the equation Anthocyanin = (A530 − 0.25 × A657) × 0.1 M–1.

SPSR soluble sugar contents and components were detected by high performance liquid chromatography (HPLC, PAL-1, Guangzhou Atago Scientific Instrument Co., LTD, Guangzhou, China) according to Xu [35].

Starch contents were determined by using an starch assay kit (Megazyme, Bray, Ireland). The starch granules were scanned and imaged using a scanning electron microscope (FEI Teneo VS, FEI Company, Hillsboro, OR, USA). Malvern laser particle size analyzer (Matersizer 3000, Malvern Instruments Ltd., Worcestershire, UK) was used to detect starch particles distribution of SPSR. The crystallinity of sweetpotato starch was analysized using an X-ray diffractometer (PANalytical, Municipality, The Netherlands).

Sweetpotato starch was extracted by the water washing precipitation method. An amount of 250 g of fresh sweetpotato storage roots were washed and then crushed with a crusher. An amount of 500 mL of distilled water was added to the crushed residue and mixed well, then filtered through gauze. The crushed residue was washed repeatedly with distilled water and the filtrate was then collected. The supernatant was removed after precipitation, and the starch repeatedly washed with distilled water until the supernatant clarifies. The wet starch was then placed in a freeze dryer to dry. The rapid viscosity analysis (RVA) method, with reference to Li [34], was used to analyze SPSR starch gelatinization characteristics. We accurately weighed 3.0 g sweetpotato starch into a RVA gelatinizing box and added 25 mL ddH2O to make the concentration of starch solution (10.7%). The RVA program setting was as follows: RVA rotor at 960 r/min speed of 10 s and then at 160 r/min speed until the end of the experiment. The initial temperature was 50 °C for 1 min, then it rose to 95 °C for 4.5 min after 4 min, and then dropped to 50 °C for 3 min after 4 min; the whole process lasted 16.7 min. Three replicates were taken for each sample. Starch pasting temperature, final viscosity, peak viscosity, and disintegration value were analyzed with RVA data analysis software (Thermocline for Windows; Version TCW3.0).

The physiological sweetness of each SPSR was calculated according to the method of Shen [3] and Li [34]. Physiological sweetness = sucrose content × 1.0 + fructose content × 0.7 + glucose content × 0.5 + maltose content × 0.25. Sweetness was measured using a portable saccharimeter (Guangzhou Suwei Electronic Technology Co., Ltd., Guangzhou, China, SW-35T) according to the instrument manual. Different sweetpotato storage roots were steamed, and the samples were randomly numbered. Samples were provided into 5 separately isolated and trained evaluators. They were asked to clean the mouth with clean water after evaluating each sample and then all samples were evaluated in turn. Samples were scored for sweetness, fiber content, taste, and total score. Finally, the average value is used as the final score.

### 4.6. Gene Expression Related to Starch and Sucrose Regulatory Networks Analysis

The transgenic lines and WT were planted in the same community, with 20 plants per variety in each community, and 3 replicates were set up. Sampling began 130 days after planting, and 3 plants were dug out from each community, making a total of 9 plants. The obtained storage roots were used to extract RNA from storage roots and reverse transcribe them into cDNA as the amplification template for RT-qPCR. Gene sequences related to starch and sucrose metabolism in sweetpotato were searched from NCBI (https://www.ncbi.nlm.nih.gov/), and RT-qPCR primers were designed using this as a template using the online tool primer 3.0 plus (https://www.primer3plus.com/), seeing Table S2 for specific primer sequences. At the same time, the running program was set up according to 2 × Q3 SYBR qPCR Master Mix (Tolo Biotech Co., Ltd., Shanghai, China) instructions, using *IbARF* as the internal reference gene.

### 4.7. Statistical Analysis

Data statistics and variance analysis were performed using SPPS20.0 (IBM, New York, NY, USA) software, and graphs were drawn and assembled using Origin8.0 (OriginLab corporation, Northampton, MA, USA) and PhotoshopC5 (Adobe Systems Incorporated, San Jose, CA, USA) software. All experiments in this article were performed using at least three independent biological experiments.

## 5. Conclusions

In summary, the experimental results showed that interference with the expression of *Ibα-XYL1* decreased starch granule size and increased soluble sugar content to improve steamed SPSR taste, but it had no significant effect on the expansion characteristics and anthocyanin content of SPSR. *Ibα-XYL1* interfered expression could up-regulate the expression of genes related to starch synthesis modifications (*GASS*, *SBE*) and starch decomposition (*AMY* and *BAM*), reduce the starch granule size, reduce the starch pasting temperature, promote the conversion of starch to maltose, increase the soluble sugar content, and improve the sweetness and taste of steamed transgenic SPSR. The results have guiding significance for sweetpotato quality improvement.

## Data Availability

The data presented in this study are available on request from the corresponding author.

## Supplementary Materials

The following supporting information can be downloaded at: https://www.mdpi.com/article/10.3390/ijms26115015/s1.
