# Supplementary material for: Ibα-XYL1 Interfered Expression Decreases Starch Granule Size and Increases Soluble Sugar Content to Improve Steamed Sweetpotato Storage Root Taste
Source: Int J Mol Sci. 2025 May 23;26(11):5015. doi: 10.3390/ijms26115015 (PMC12154300; doi:10.3390/ijms26115015)
Supplement: Supplementary file 1 [file ijms-26-05015-s001.zip › ijms-3558814-supplementary.pdf]

Supplementary material

Table S1 Ibα-XYL1 interacting protein information from CO-IP-MS

Accession	Gene	Length	log2 XYL/WT	Diff Sig	Description	Functional annotation
G8XR51	SuS	727	1.07	+	Sucrose synthase	Sucrose-cleaving enzyme that provides UDP-glucose and fructose for various metabolic pathways
L8AYL4	IbNINV1	308	1.09	+	Alkaline/neutral invertase	Invertase that cleaves sucrose into glucose and fructos
A0A5H2BGA9	FBA	357	-1.85	-	Fructose-bisphosphate aldolase	Carbohydrate degradation
U3QZN5	rbcL	480	-1.89	-	Ribulose bisphosphate carboxylase large chain	The carboxylation of D-ribulose 1,5-bisphosphate, the primary event in carbon dioxide fixation, as well as the oxidative fragmentation of the pentose substrate in the photorespiration process
A0A0H5ASB3	rbcS2	167	-4.04	-	Ribulose bisphosphate carboxylase small subunit	The carboxylation of D-ribulose 1,5-bisphosphate, the primary event in carbon dioxide fixation, as well as the oxidative fragmentation of the pentose substrate.
Q4PLT2	SBE	214	-3.11	-	Starch branching enzyme	Starch metabolic process
Q8W517	SPS	1048	-1.20	-	Sucrose-phosphate synthase	Plays a role in photosynthetic sucrose synthesis by catalyzing the rate-limiting step of sucrose biosynthesis from UDP-glucose and fructose- 6-phosphate.
Q94C05	Ibbfruct3	661	-1.41	-	Beta-fructofuranosidase	Carbohydrate metabolic process
S4X741	UDPGH10	480	-1.44	-	UDP-glucose 6-dehydrogenase	UDP-glucuronate biosynthetic process
A0A142KXI7		131	4.16	+	OPR2a	Oxidoreductase activity
A0A142KXJ3	13-LOX	910	2.85	+	Lipoxygenase	Oxylipin biosynthetic process
M4T7S0	PLD	146	2.91	+	Phospholipase D phospholipid catabolic process	Phospholipid catabolic process
K9P1V0	TRX	122	3.66	+	Thioredoxin	Protein-disulfide reductase activity
K0II87		439	-2.47	-	Chloroplast ribulose bisphosphate carboxylase	Rribulose-1,5-bisphosphate carboxylase/oxygenase activator activity
Q9ZP19	co-1	496	-2.82	-	Polyphenol oxidase I	Copper ion binding
K9P2L0	GPX	169	0.59	+	Glutathione peroxidase	Response to oxidative stress

Table S2 Primer sequence

ID	primer	Gene lander
IbAMY	ATTCTTCAGGCTGCTGTGCT<br>CTCCGGGATGGGTGAGGATA	KT281131.1
IbBAM	CGGGAAGAGACGGTTATCGG<br>TGCATTCTCACCTGCCACAT	D12882.1
IbSS	ATTCCCTGGACACCTTGCAG<br>TGCACATCTTGGCCCATCAT	AF068834.1
IbSBE	CGCCTTCCTGATGGTTCAGT<br>GTACTGGTGTTCTGCCGTCA	AB042937.1
IbAGPase	GAGATATCCCACATCCAACGACTT<br>TAGGGCCAAGTTAGCGTCGTAG	AJ252316.1
IbGBSS	GACTGCGGCATCACTGGTATTT<br>GAACTTAGAAATCGCAGCAT	AB071604
IbSuS	GTTTCTAAGCTGGACCGCCT<br>AGTTCACCAGGTTCGCAGAG	itf11g07860.t1
IbSuPS	TGCAGATGCTGGTGACTCTG<br>AGCAAGCTCCCTCAATGGAC	itf03g21140.t1
IbARF	CTTTGCCAAGAAGGAGATGC<br>TCTTGTCCTGACCACCAACA	JX177359
Ibα-XYL1	GAACCCCAGTTGCAGATACTTC<br>AAGCAGTGATGCCCATATGC	
RNAi-XYL-check	CCCAAAGATGGACCCCCA<br>GAACCCGTTTGGGTGAGC	
Bar	TACCGGCAGGCTGAAGTC<br>AAATCGAATCGATGAGCCCAGAA	

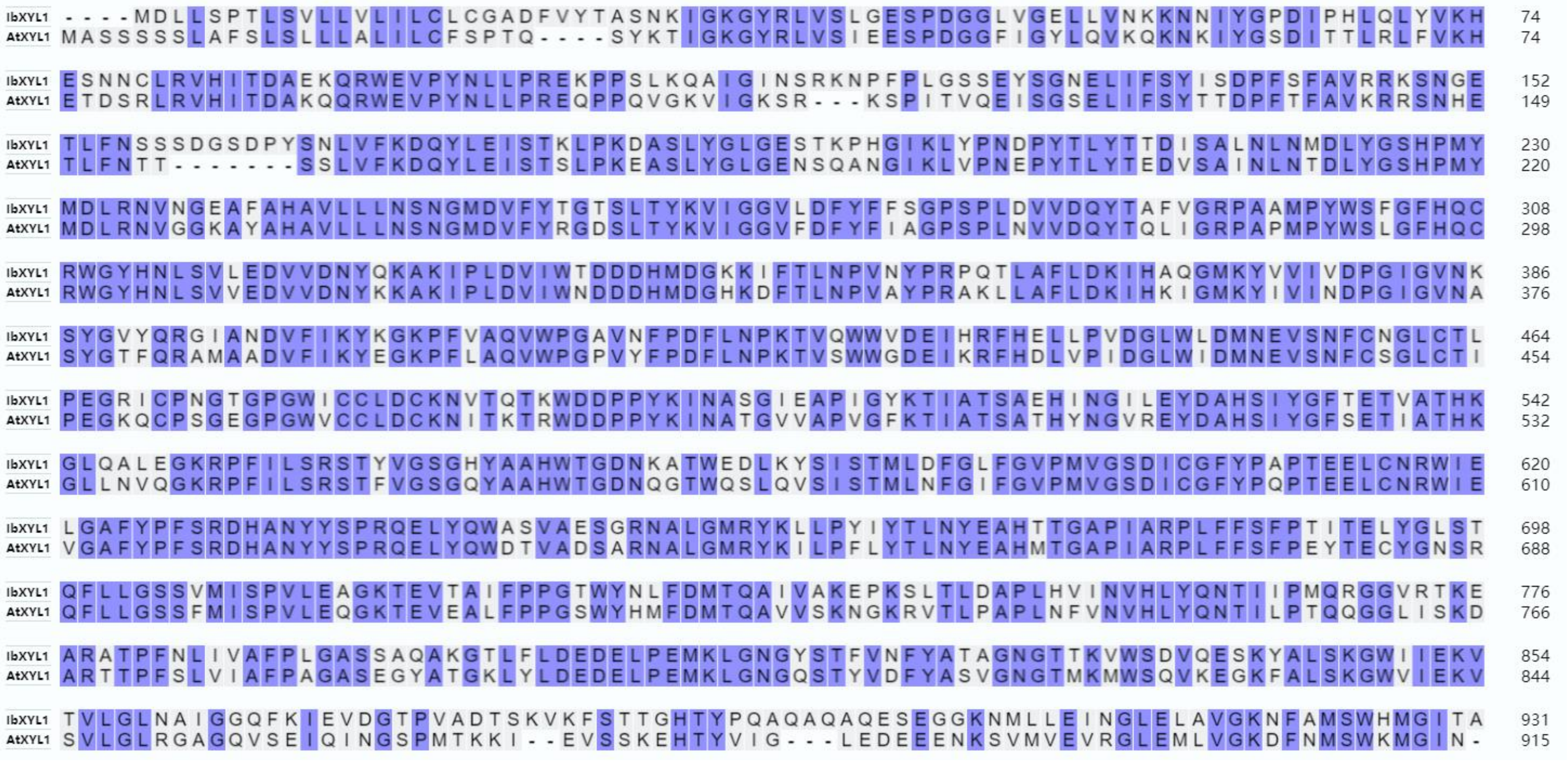

Fig S1 Amino acid sequence comparison of xylosidase from sweetpotato and *Arabidopsis thaliana*

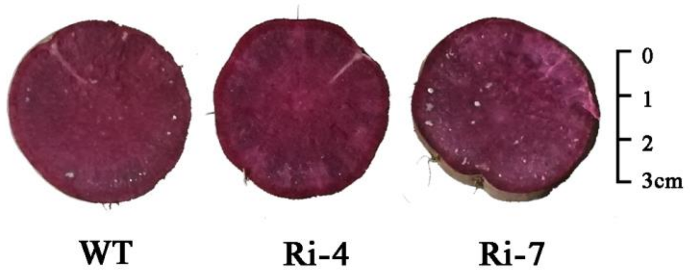

Fig S2 Cross-section observation of *Ibα-XYL1* interfered with transgenic SPSR

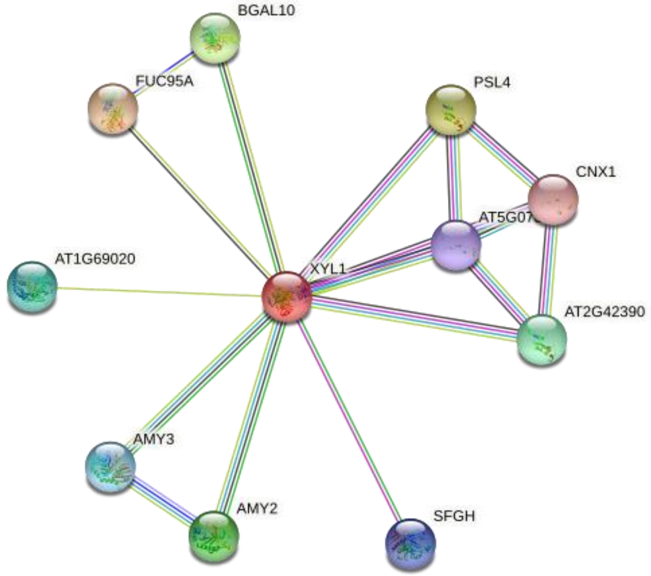

Fig S3 Prediction of *Arabidopsis* Atα-XYL1 interacting proteins

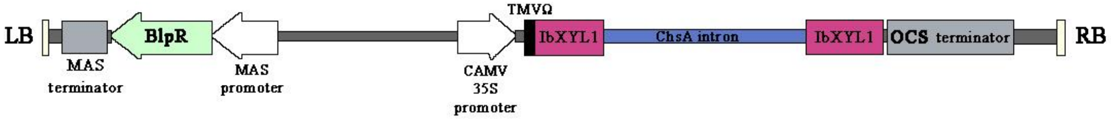

Fig S4 Structure diagram of *Ibα-XYL1* gene RNA interference expression vector

Sequence S1 The target sequences for RNAi-Ibα-XYL1

ATGGACTTACTTTCTCCCACTCTTTCTGTGCTTCTAGTTTTGATTCTTTGTTTATGTGGTGCTGATTTTGTATACACAGCATCAAACAAGATTGGGAAAGGCTACCGTTTGGTCTCCCTGGGAGAGTCCCC

TGATGGTGGCCTTGTGGGCGAACTCCTTGTCAACAAGAAAAACAATATCTATGGCCCTGATATTCCTCATTTGCAGCTTTATGTTAAACATGAATCAAATAATTGCTTGAGGGTTCACATTACAGATGCAG

AGAAGCAGAGATGGGAAGTCCCGT (***Ibα-XYL1*** **)**

GTAAGAATTTCTTATGTTACATTATTACATTCAACGTTTTATCTTAATTGGCTCTTCATTTGATTGAAATTTGACAATTATTTCTTGTTTTTTTTTTTGTCACACTCTTTTTGGGTTGGGGTGGCCGACGAATT

GTGGGAAGGTAGAAAGAGGGGAGGACTTTTGTTATACTCCATTAGTAATTACTGTTTCCGTTTCAATTTATGTGACAATATTTCCTTTTTAGTCGGTTCCAAAAGAAAATGTCAGCATTATAAACAATTTAA

TTTTGAAATTACAATTTTGCCATTAATAAAATGATTTACAACCACAAAAGTATCTATGAGCCTGTTTGGGTGGGCTTATAAGCAGCTTATTTTAAGTGGCTTATAAGTCAAAAAGTGACANTTTTTGAGAAG

TTAGAAAATCCTAACTTCTCAAAAAGTAGCTTTTAAGCCACTTATGACTTATAAGTCCAAAAATTTTTAAGTTACCAAACATATATTAATGGGTTTATAAGCTTATAAGCCACTTTTAAGCTCACCCAAACGG

GTTCTATGTCTCACTTTAGACTACAAATTTTAAAAGTCTTCATTTATTTCTTAATCTCCGTGGCGAGTNAAACTATAACACATAAAGTGAAACGGAGGGAATAAGATGGAGTCATAAACTAATCCAAATCTAT

ACTCTCTCCGTTAATTTGTTTTTTAGTTTGATTTGGTACATTAATAAAACAGATTTTTCGAAGGTTATAAACACAGACAGATGTTTCCCAGCGAGCTAGCAAAATTCCAAGATTTCTGTCGAAAATTCGTGT

GTTTCTAGCTAGTACTTGATGTTATCTTTAACCTTTTAGTAATTTTTTGTCCTTTTCTTTCTATTTTTCATCTTACAATGAATTATGAGCAAGTTCCTTAAGTAGCATCACACGTGAGATGTTTTTTATGATATT

GACTAAATCCAATCTTTACCATTCCTTAACTAGTAAAATACAACACATGTTAATTGATACATTGCTTAACACTGAGGTTAGAAAATTTTAGAAATTAGTTGTCCAAATGCTTTGAAATTAGAAATCTTTAATCC

CTTATTTTTTTTTAAAATGTTTTTTCTCACTCCAAAGAAAGAGAAACTGACATGAAAGCTCAAAAGATCATGAATCTTACTAACTTTGTGGAACTAAATGTACATCAGAATGTTTCTGACATGTGAAAATGA

AAGCTCTTAATTTTCTTCTTTTATTTATTGAGGGTTTTTGCATGCTATGCATTCAATTTGAGTACTTTAAAGCACCTATAAACACTTACTTACACTTGCCTTGGAGTTTATGTTTTAGTGTTTTCTTCACATCT

TTTTTGGTCAATTTGCAGGTATTTGGATCC (Intron)

GGGACTTCCCATCTCTGCTTCTCTGCATCTGTAATGTGAACCCTCAAGCAATTATTTGATTCATGTTTAACATAAAGCTGCAAATGAGGAATATCAGGGCCATAGATATTGTTTTTCTTGTTGACAAGGAGT

TCGCCCACAAGGCCACCATCAGGGGACTCTCCCAGGGAGACCAAACGGTAGCCTTTCCCAATCTTGTTTGATGCTGTGTATACAAAATCAGCACCACATAAACAAAGAATCAAAACTAGAAGCACAGAA

AGAGTGGGAGAAAGTAAGTCCAT (***Ibα-XYL1*** **)**

Sequence S2 The sequences of *Ibα-XYL1*

ATGGACTTACTTTCTCCCACTCTTTCTGTGCTTCTAGTTTTGATTCTTTGTTTATGTGGTGCTGATTTTGTATACACAGCATCAAACAAGATTGGGAAAGGCTACCGTTTGGTCTCCCTGGGAGAGTCCCC

TGATGGTGGCCTTGTGGGCGAACTCCTTGTCAACAAGAAAAACAATATCTATGGCCCTGATATTCCTCATTTGCAGCTTTATGTTAAACATGAATCAAATAATTGCTTGAGGGTTCACATTACAGATGCAG

AGAAGCAGAGATGGGAAGTCCCATATAACCTTTTACCCAGAGAAAAGCCTCCATCTTTGAAACAAGCCATTGGTATTAATTCAAGAAAGAACCCTTTTCCACTTGGGAGCTCAGAGTATTCTGGGAATGA

GCTCATCTTTAGCTACATCTCTGACCCTTTTAGCTTTGCTGTGAGAAGAAAATCAAATGGGGAGACCCTTTTCAATTCCAGCTCTGATGGATCCGACCCGTATAGCAATTTGGTTTTCAAGGACCAGTACC

TTGAAATATCCACCAAATTGCCTAAAGATGCTTCACTGTATGGGCTTGGGGAGAGCACAAAGCCCCATGGGATAAAGCTGTACCCTAATGACCCTTACACCCTCTACACCACTGATATATCAGCCCTTAAT

CTGAATATGGACTTGTATGGGTCCCACCCTATGTACATGGATTTGAGGAATGTGAATGGGGAGGCATTTGCCCATGCAGTCCTCTTGCTCAACAGCAATGGGATGGATGTGTTCTACACTGGGACTTCTT
TGACATACAAAGTGATTGGGGGTGTTTTGGACTTTTACTTCTTCTCCGGTCCCTCTCCTCTTGATGTTGTTGATCAGTACACCGCCTTTGTAGGCAGGCCAGCTGCAATGCCGTATTGGTCTTTTGGGTTC
CATCAATGTAGATGGGGTTACCACAATTTGTCTGTTCTTGAGGATGTTGTTGATAACTATCAGAAGGCGAAAATCCCTCTTGATGTGATTTGGACTGATGATGATCACATGGATGGAAAGAAGATCTTTAC
CCTCAATCCAGTGAACTATCCTCGCCCACAGACATTGGCATTCCTAGATAAAATTCATGCACAAGGCATGAAATACGTTGTCATTGTAGATCCTGGAATTGGTGTTAACAAAAGTTATGGAGTCTACCAAA
GAGGTATAGCCAATGATGTGTTCATCAAGTATAAGGGCAAGCCATTTGTGGCCCAAGTATGGCCAGGAGCTGTTAATTTCCCTGACTTCCTTAACCCAAAGACTGTTCAATGGTGGGTTGATGAAATTCAT
CGATTCCATGAACTTTTACCGGTTGATGGGCTTTGGCTTGACATGAATGAAGTTTCCAACTTCTGTAATGGTTTGTGCACACTACCCGAGGGTAGGATTTGCCCTAATGGAACTGGACCCGGTTGGATCT
GCTGCCTAGACTGTAAGAATGTAACACAAACCAAATGGGATGATCCACCTTACAAGATCAACGCTTCTGGAATAGAGGCACCTATAGGATACAAAACCATTGCTACAAGTGCAGAACACATCAATGGAATT
TTGGAGTATGATGCCCACAGCATTTATGGTTTCACCGAGACTGTTGCCACTCACAAAGGCCTTCAAGCACTTGAGGGCAAGCGGCCATTTATATTATCCCGCTCTACATATGTTGGTTCTGGCCACTATGC
TGCTCACTGGACAGGGGATAACAAGGCAACTTGGGAAGATTTGAAGTATTCAATTTCCACAATGCTGGATTTTGGATTATTTGGTGTGCCTATGGTCGGGTCAGATATATGTGGATTCTATCCAGCACCTA
CAGAAGAGCTGTGCAACCGCTGGATTGAACTAGGTGCTTTCTATCCTTTTTCAAGAGATCATGCTAACTACTATTCTCCTCGACAGGAGCTCTATCAGTGGGCTTCAGTTGCTGAATCTGGACGTAATGCT
TTGGGTATGAGATATAAGCTTCTTCCATATATTTACACTTTGAACTATGAAGCACATACGACTGGGGCACCAATTGCTAGACCACTATTCTTCTCTTTCCCAACTATAACTGAACTATATGGATTGAGCACTC
AGTTCTTGCTAGGAAGCAGTGTCATGATCTCTCCAGTGCTGGAAGCGGGCAAAACTGAGGTCACAGCTATTTTCCCTCCGGGCACTTGGTACAATCTGTTCGATATGACACAGGCCATTGTTGCAAAGG
AGCCAAAGTCCTTGACACTCGATGCACCTTTGCATGTGATCAACGTGCATTTGTATCAGAATACCATAATTCCTATGCAGCGTGGTGGAGTGAGAACTAAAGAAGCACGAGCAACCCCATTCAACCTTATA
GTTGCCTTCCCATTGGGGGCTTCTAGTGCACAAGCCAAAGGAACTCTCTTCCTTGATGAGGATGAGCTCCCTGAAATGAAGCTTGGAAATGGCTACTCAACATTTGTAAATTTCTATGCAACAGCGGGCA
ATGGAACCACTAAGGTATGGTCAGATGTTCAGGAAAGCAAGTATGCATTGAGTAAAGGGTGGATCATAGAGAAAGTAACTGTATTAGGGTTGAATGCAATTGGGGGGCAGTTCAAAATTGAAGTTGATGG
AACCCCAGTTGCAGATACTTCCAAGGTGAAGTTTAGCACAACCGGACATACATATCCTCAGGCTCAGGCTCAGGCACAGGAAAGCGAAGGAGGTAAGAACATGCTGCTTGAGATTAATGGATTGGAGTT
GGCTGTAGGTAAAAACTTTGCCATGTCATGGCATATGGGCATCACTGCTTAG
